# Development of a predictive model integrating urogenital pathogens and inflammatory markers for human papillomavirus infection

**DOI:** 10.3389/fcimb.2026.1741813

**Published:** 2026-04-13

**Authors:** Jingxi Zhang, Biyao Jiang, Juan Tao, Mengyu Chen, Songshu Xiao, Yafang Gong

**Affiliations:** 1 Department of Obstetrics and Gynecology, Xiangya Third Hospital, Central South University, Changsha, Hunan, China; 2 Department of Maternal and Child Health, Xiangya School of Public Health, Central South University, Changsha, Hunan, China; 3 Department of Gynecology Laboratory, Xiangya Third Hospital, Central South University, Changsha, Hunan, China

**Keywords:** *Human papillomavirus* (HPV), *Chlamydia trachomatis* (CT), *Ureaplasma urealyticum* (UU), *Ureaplasma parvum* (UP), *Mycoplasma hominis* (MH), vaginal microecology, co-infection, logistic regression

## Abstract

**Objective:**

The objective of the present study was to evaluate the relationship between common urogenital pathogens and human papillomavirus (HPV) infection and to assess the impact of coinfections on vaginal microecology.

**Methods:**

This hospital-based cross-sectional study included 330 reproductive-aged women. Multiplex polymerase chain reaction (PCR) was applied to detect *Chlamydia trachomatis* (CT), *Neisseria gonorrhoeae* (NG), *Ureaplasma urealyticum* (UU), *Ureaplasma parvum* (UP), *Mycoplasma hominis* (MH), and *Mycoplasma genitalium* (MG). HPV DNA genotyping was also performed. Clinical characteristics and laboratory results were evaluated using univariate and multivariate logistic regression analyses to identify independent risk factors for HPV infection. A logistic regression model was constructed, and its predictive performance was evaluated by receiver operating characteristic (ROC) curve and calibration analyses.

**Results:**

The overall HPV positivity rate was 46.4%. Univariate analysis revealed associations of HPV infection with CT, NG, UU, UP, MH, elevated leukocyte count, and vaginitis (all *P* < 0.05). Multivariate analysis revealed that CT infection (aOR=4.115), UU infection (aOR=3.937), elevated leukocyte count (aOR=2.076), and abnormal vaginal discharge (aOR=2.987) were independent predictors for HPV. The prediction model demonstrated good discrimination (AUC≈0.85). In addition, UU, MH, CT, and UP infections were significantly linked to bacterial vaginosis and other vaginal disorders.

**Conclusions:**

CT and UU infections are strongly associated with HPV infection. Elevated leukocyte count and abnormal vaginal discharge serve as inflammatory indicators that increase prediction accuracy. The proposed model, which integrates pathogen detection and host inflammatory status, performs well in identifying women at high risk of HPV, thereby supporting early screening and cervical cancer prevention.

## 1 Introduction

HPV is among the most common sexually transmitted pathogens worldwide. Globally, among women with normal cervical cytology (NCC), earlier reports showed an HPV prevalence of 10.4% and 11.7% in 2007 and 2010, respectively, which was adjusted to 9.9% in 2019 (de Sanjosé et al., 2007; Bruni et al., 2010; Zhang et al., 2010). In China, the prevalence of cervical HPV in a study of 427,401 women aged 20 years or older in 2021 was 15.0% and high-risk HPV was 12.1% (Bao et al., 2021). Persistent infection is the major cause of cervical cancer and cervical intraepithelial neoplasia (CIN) (Liang et al., 2019; Yang et al., 2024). Although most HPV infections resolve spontaneously within 1–2 years, a subset of women develop persistent infections that may progress to CIN and invasive cancer. The determinants of HPV persistence are multifactorial and involve viral, host, and environmental factors. Among these, disturbances in vaginal microecology and coinfections with other genital pathogens have received increasing attention (Laniewski et al., 2020; Huang et al., 2024).

Recent studies have suggested that common urogenital pathogens, such as *Chlamydia trachomatis* (CT), *Ureaplasma urealyticum* (UU), *Mycoplasma hominis* (MH), and bacterial vaginosis (BV), may facilitate HPV acquisition and persistence by disrupting epithelial barriers, inducing inflammatory responses, and altering the local immune microenvironment (Watts et al., 2005; Guo et al., 2012; Zadora et al., 2019). In particular, recent evidence has indicated that the composition of the cervicovaginal microbiome, including depletion of Lactobacillus and enrichment of anaerobes such as *Gardnerella* and *Prevotella*, may increase the persistence of HPV through immune modulation, oxidative stress, and metabolic reprogramming, thereby contributing to CIN and cervical carcinogenesis (Huang et al., 2024). In addition to microbial dysbiosis, host inflammatory markers such as elevated leukocyte count have been implicated in HPV persistence and cervical disease progression. Leukocytosis reflects local inflammatory activation, which may impair mucosal barrier integrity and antiviral immunity, thereby creating a microenvironment conducive to viral persistence and neoplastic transformation (Castle et al., 2001; Madeddu et al., 2022; Shih et al., 2024). Epidemiological studies and meta-analyses have further demonstrated significant associations of CT, UU, and BV with HPV persistence and CIN progression (Gillet et al., 2011; Camporiondo et al., 2016; Liang et al., 2019; Zhong et al., 2023; Yang et al., 2024).

However, most previous investigations have focused on single pathogens, with limited attention given to polymicrobial coinfections, host inflammatory status, and their combined impact on HPV infection. Moreover, few attempts have been made to integrate these factors into predictive models with potential clinical utility. Therefore, the present study aimed to evaluate the associations between HPV infection and common urogenital pathogens, including CT, NG, UU, UP, MH, and MG, and to assess the predictive value of leukocyte count and vaginitis. On the basis of these findings, we constructed and validated a logistic regression model for HPV infection, with the goals of improving the early identification of women with a high risk of HPV and supporting cervical cancer prevention strategies.

## 2 Methods and materials

### 2.1 Clinical data

**Figure 1:**
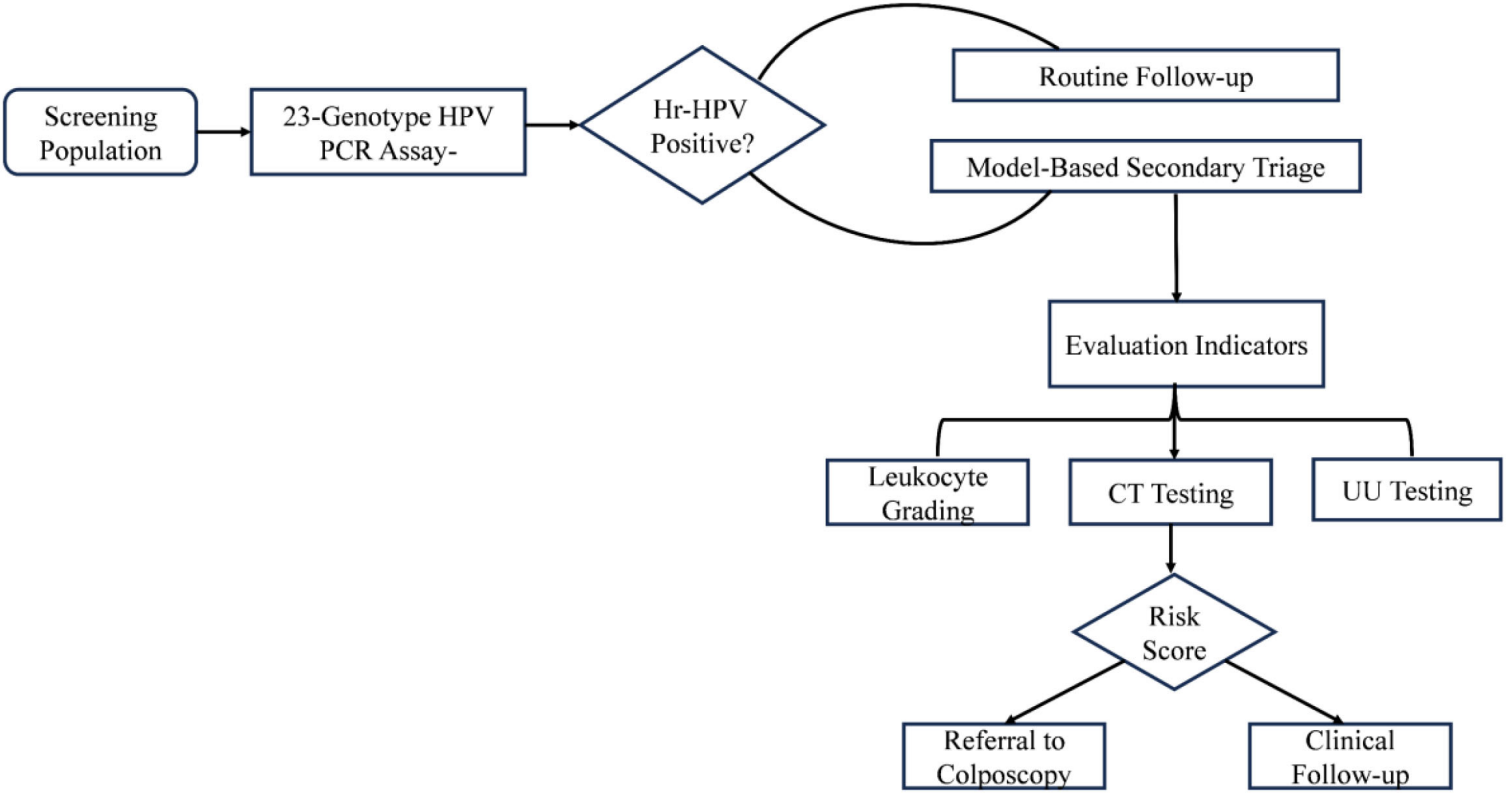
Proposed clinical workflow for cervical cancer screening using 23-genotype HPV primary screening and model-based secondary triage.

The present hospital-based cross-sectional study included 330 women aged 18–65 years who were recruited from gynecological outpatient clinics and routine health screening centers. All patient demographic and clinical information was retrieved securely from the hospital’s electronic medical records system and kept strictly confidential. Based on standardized clinical and microbiological diagnostic criteria, the participants were categorized into specific vaginal microecology groups (including healthy controls and various types of vaginitis). Furthermore, patients were divided into HPV-positive and HPV-negative groups according to their subsequent HPV DNA genotyping results (**Figure 1**).

The inclusion criteria were as follows: (1) reproductive age (18–65 years); (2) no menstruation or sexual intercourse within 48 hours before sampling; and (3) no systemic antibiotic, antifungal, or antiviral therapy within the previous 2 weeks. The exclusion criteria included pregnancy, immunodeficiency disorders, a history of cervical neoplasia, or incomplete clinical data. Written informed consent was obtained from all participants, and the study was approved by the institutional ethics committee. For patients who consented to sample collection during several visits, only those whose data were collected during the first visit were selected and included in the present study.

### 2.2 Sample collection and laboratory analysis

Vaginal and endocervical swabs were collected during standardized pelvic examinations by experienced gynecologists using aseptic techniques. The samples were transported at 4 °C and processed within 2 hours to ensure integrity. The detection of CT, NG, UU, UP, MG, and MH DNA was performed using a commercially validated *in vitro* diagnostic (IVD) kit (Urogenital Pathogen Nucleic Acid Detection Kit, Wuhan Mingde Biological Technology Co., Ltd., Wuhan, China) based on multiplex real-time PCR combined with fluorescent probe technology. This NMPA-registered assay has established analytical sensitivity, specificity, and clinical validation per the manufacturer’s specifications. Amplification and analysis were conducted using the SLAN-96S Automatic Medical PCR Analysis System (Shanghai Hongshi Medical Technology Co., Ltd., Shanghai, China). Following the manufacturer’s guidelines, the test result was interpreted as positive if the amplification curve exhibited exponential growth with a cycle threshold (Ct) value ≤ 40, and negative if there was no exponential growth or the Ct value was > 40. Cervical cytology was evaluated using the ThinPrep liquid-based cytology system (Hologic, USA). HPV DNA detection and genotyping were performed using a validated commercial assay (23 HPV Classification Test Kit, Hybribio, China). Rather than sequencing, this assay utilizes multiplex polymerase chain reaction (PCR) combined with flow-through hybridization to identify common high-risk and low-risk subtypes. Each batch included positive and negative controls, and all tests were performed in duplicate to ensure reproducibility.

In addition, vaginal smears were prepared and examined microscopically at 400× magnification to assess the leukocyte count. The number of leukocytes per high-power field (HPF) was recorded, and the mean count was calculated across 10 consecutive fields. An average of >10 leukocytes/HPF was defined as leukocytosis and was used as an indicator of local inflammatory status.

### 2.3 Clinical diagnosis

All diagnoses were established through standardized clinical assessment and confirmatory laboratory testing. BV was diagnosed using Amsel’s criteria (requiring ≥3 of homogeneous discharge, pH >4.5, positive amine test, and clue cells). VVC was confirmed by microscopic visualization of pseudohyphae or budding yeast cells. AV diagnosis required both quantitative aerobic bacterial overgrowth (>10^3 CFU/mL) and inflammatory markers (e.g., elevated leukocytes). TV was identified by direct microscopic observation of motile *Trichomonas vaginalis* trophozoites. CV was identified by the cytolysis of vaginal epithelial cells with predominant *Lactobacillus* morphotypes. Mixed infections were defined by the concurrent detection of multiple pathogens with corresponding clinical manifestations.

### 2.4 Statistical analysis

All the statistical analyses were conducted using SPSS software (version 27.0). Categorical variables are presented as frequencies and proportions, with group comparisons performed using Pearson’s chi-square test or Fisher’s exact test, as appropriate. Continuous variables were analyzed using independent Student’s t tests for normally distributed data or the Mann–Whitney U test for nonparametric distributions. Associations between coinfections and HPV status were evaluated using univariate and multivariate logistic regression. The multivariate model was adjusted for clinically relevant confounders (including leukocyte count and abnormal vaginal discharge). A logistic regression model was developed, and its performance was evaluated by the area under the ROC curve (AUC) and Hosmer–Lemeshow test. The results are reported as odds ratios (ORs) or adjusted odds ratios (aORs) with 95% confidence intervals (CIs). A two-tailed P value <0.05 was considered to indicate statistical significance.

## 3 Results

### 3.1 Patient characteristics

A total of 330 women aged 18–65 years (mean age 36.2 ± 8.5 years) were included in the present study. Among the participants, 153 (46.4%) women were HPV positive, and 177 (53.6%) women were HPV negative. The detection rates of urogenital pathogens were as follows: CT in 79 (23.9%) participants, NG in 9 (2.7%) participants, UU in 208 (63.0%) participants, UP in 185 (56.1%) participants, MG in 9 (2.7%) participants, and MH in 114 (34.5%) participants. Additionally, 183 women (55.5%) were diagnosed with vaginal infections, including BV (n = 108), VVC (n = 10), AV (n = 2), TV (n = 1), CV (n = 6), and mixed infections (n = 20). No significant difference in age was observed between the HPV-positive and HPV-negative groups (*P* > 0.05). Overall, the most prevalent pathogen was UU, followed by UP and MH.

### 3.2 Urogenital pathogen distribution profiles across different diagnostic groups

The prevalence of CT, UU, UP, and MH was significantly greater in HPV-positive women than in HPV-negative women (all *P* < 0.001). Although NG infection was relatively uncommon, its prevalence differed significantly between the two groups (*P* = 0.014). In contrast, MG infection demonstrated only a borderline association with HPV status (*P* = 0.051). Moreover, HPV-positive women exhibited a significantly higher incidence of overall vaginitis compared to HPV-negative women (**Table 1**).

**Table 1:** Comparative distribution of urogenital pathogens across various diagnostic groups.

	CT N=79	NG N=9	UU N=208	UP N=185	MG N=9	MH N=114	Vaginitis N=183
HPV positive (N = 153)	65 (42.5%)	8 (5.20%)	129 (84.3%)	101 (66.0%)	7 (4.60%)	81 (52.9%)	125 (81.7%)
HPV negative (N = 177)	14 (7.90%)	1 (0.60%)	79 (44.6%)	84 (47.5%)	2 (1.10%)	33(18.6%)	58 (32.8%)

CT, Chlamydia trachomatis; NG, Neisseria gonorrhoeae; UU, Ureaplasma urealyticum; UP, Ureaplasma parvum; MG, Mycoplasma genitalium; MH, Mycoplasma hominis; HPV, human papillomavirus; Vaginitis, diagnosed cases of vaginitis (including BV, VVC, AV, TV, and CV).

### 3.3 Univariate analysis of the association between HPV infection and multiple pathogens

Univariate logistic regression analysis revealed that the leukocyte count (OR = 2.630, 95% CI: 1.968–3.515, *P* < 0.001), CT infection (OR = 8.600, 95% CI: 4.567–16.201, *P* < 0.001), NG infection (OR = 11.000, 95% CI: 1.378–87.847, *P* = 0.024), UU infection (OR = 6.668, 95% CI: 3.934–11.294, *P* < 0.001), UP infection (OR = 2.150, 95% CI: 1.376–3.363, *P* < 0.001), MH infection (OR = 4.889, 95% CI: 2.989–7.996, *P* < 0.001), and vaginal discharge (OR = 9.159, 95% CI: 5.471–15.348, *P* < 0.001) were significantly associated with HPV infection. No significant association between MG infection and HPV infection was observed (OR = 4.195, 95% CI: 0.858–20.524, *P* = 0.077).

### 3.4 Multivariate analysis of the association between HPV infection and multiple pathogens

**Figure 2:**
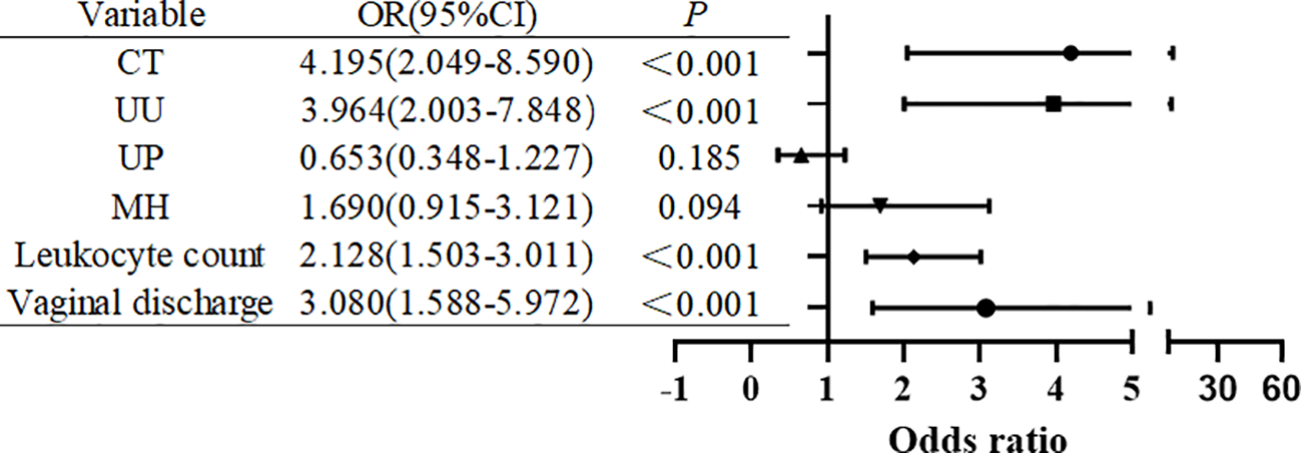
Multivariate logistic regression analysis of the associations between urogenital pathogens and HPV infection (forest plot).

Multivariate logistic regression analysis revealed that CT infection (aOR = 4.115, 95% CI: 1.993–8.501, *P* < 0.001),abnormal vaginal discharge (aOR = 2.987, 95% CI: 1.528–5.838, *P* < 0.001), elevated leukocyte count (aOR = 2.076, 95% CI: 1.461–2.947, *P* < 0.001), and UU infection (aOR = 3.937, 95% CI: 1.974–7.845, *P* < 0.001) were independently associated with HPV infection. Although NG, UP, and MH infections were significantly associated with HPV infection in the univariate analysis, they did not retain significance in the multivariate models(all *P*>0.05).The logistic regression prediction model was constructed on the basis of the above four variables and demonstrated favorable predictive performance. ROC curve analysis yielded an AUC of 0.853 (95% CI: 0.811–0.894, *P* < 0.05), indicating strong discriminative ability. The Hosmer–Lemeshow goodness-of-fit test yielded *P*>0.05, suggesting good calibration (**Table 2**, **Figure 2**).

**Table 2:** Univariate analysis of risk factors for HPV infection.

Variables	*P*	Adjusted OR	OR (95%CI)
leukocyte count	< 0.001	2.630	1.968-3.515
*Chlamydia trachomatis*	< 0.001	8.600	4.567-16.201
*Neisseria gonorrhoeae*	0.024	11.000	1.378-87.847
*Ureaplasma urealyticum*	< 0.001	6.668	3.934-11.294
*Ureaplasma parvum*	< 0.001	2.150	1.376-3.363
*Mycoplasma hominis*	< 0.001	4.889	2.989-7.996
*Mycoplasma genitalium*	0.077	4.195	0.858-20.524
vaginal discharge	< 0.001	9.159	5.471-15.348

**Figure 3:**
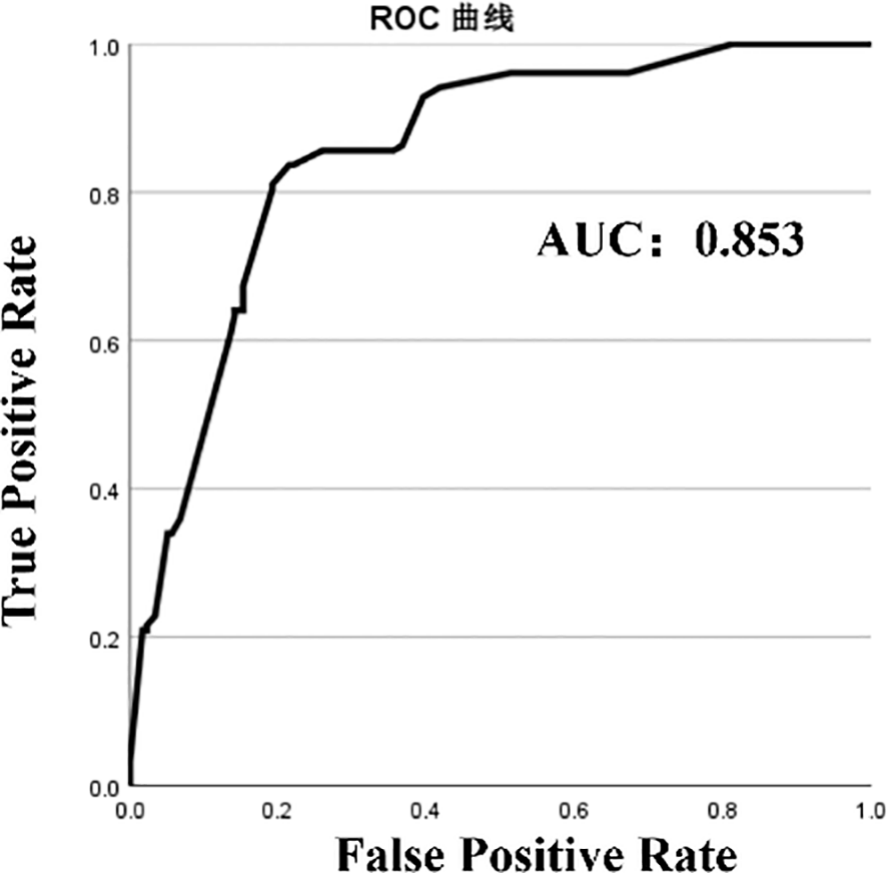
Multivariate logistic regression analysis of the associations between urogenital pathogens and vaginitis (forest plot).

Only CT infection, UU infection, and inflammatory markers (elevated white blood cell count and abnormal vaginal discharge) remained independently associated with HPV infection after adjustment, suggesting that these pathogens and related inflammatory responses are key risk factors for HPV infection. The developed prediction model demonstrated high accuracy in distinguishing high-risk populations, indicating that combining pathogen detection with clinical inflammatory markers could address the limitations associated with relying solely on HPV testing (**Figure 3**).

### 3.5 Association between urogenital pathogens and vaginitis

Univariate logistic regression analysis revealed that leukocyte count (OR = 2.264, 95% CI: 1.717–2.984, *P* < 0.001), CT infection (OR = 8.292, 95% CI: 4.085–16.820, *P* < 0.001), UU infection (OR = 9.146, 95% CI: 5.439–15.370, *P* < 0.001), UP infection (OR = 5.456, 95% CI: 3.396–8.770, *P* < 0.001), MH infection (OR = 7.854, 95% CI: 4.440–13.893, *P* < 0.001), and HPV infection (OR = 9.159, 95% CI: 5.466–15.348, *P* < 0.001) were significantly associated with vaginitis. No significant associations were detected for NG or MG infection (both *P* > 0.05).

**Figure 4:**
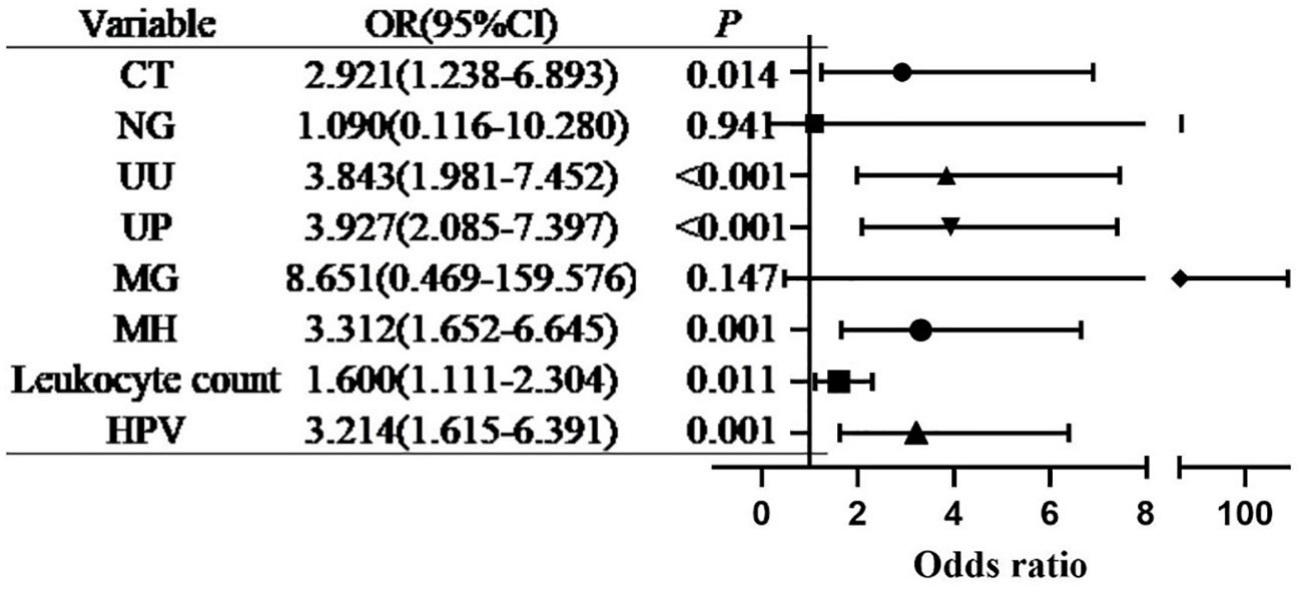
Multivariate logistic regression analysis of the associations between urogenital pathogens and vaginitis (forest plot).

Multivariate logistic regression analysis revealed that UU infection (aOR = 3.843, 95% CI: 1.981–7.452, *P* < 0.001), elevated leukocyte count (aOR = 1.600, 95% CI: 1.111–2.304, *P* = 0.011), CT infection (aOR = 2.921, 95% CI: 1.238–6.893, *P* = 0.014), UP infection (aOR = 3.927, 95% CI: 2.085–7.397, *P* < 0.001), MH infection (aOR = 3.312, 95% CI: 1.652–6.645, *P* = 0.001), and HPV infection (aOR = 3.214, 95% CI: 1.615–6.391, *P* = 0.001) were independently associated with the occurrence of vaginitis. No significant associations were detected for NG or MG infection (both P > 0.05). Details are shown in **Table 3** and **Figure 4**.

**Table 3:** Univariate analysis of risk factors for vaginitis.

Variables	*P*	Adjusted OR	OR (95%CI)
leukocyte count	< 0.001	2.264	1.717-2.984
*Chlamydia trachomatis*	< 0.001	8.292	4.085-16.820
*Neisseria gonorrhoeae*	0.075	6.674	0.825-53.984
*Ureaplasma urealyticum*	< 0.001	9.146	5.439-15.370
*Ureaplasma parvum*	< 0.001	5.456	3.396-8.770
*Mycoplasma hominis*	< 0.001	7.854	4.440-13.893
*Mycoplasma genitalium*	0.077	4.195	0.858-20.524
HPV	< 0.001	9.159	5.466-15.348

### 3.6 Association between urogenital pathogens and BV

**Figure 5:**
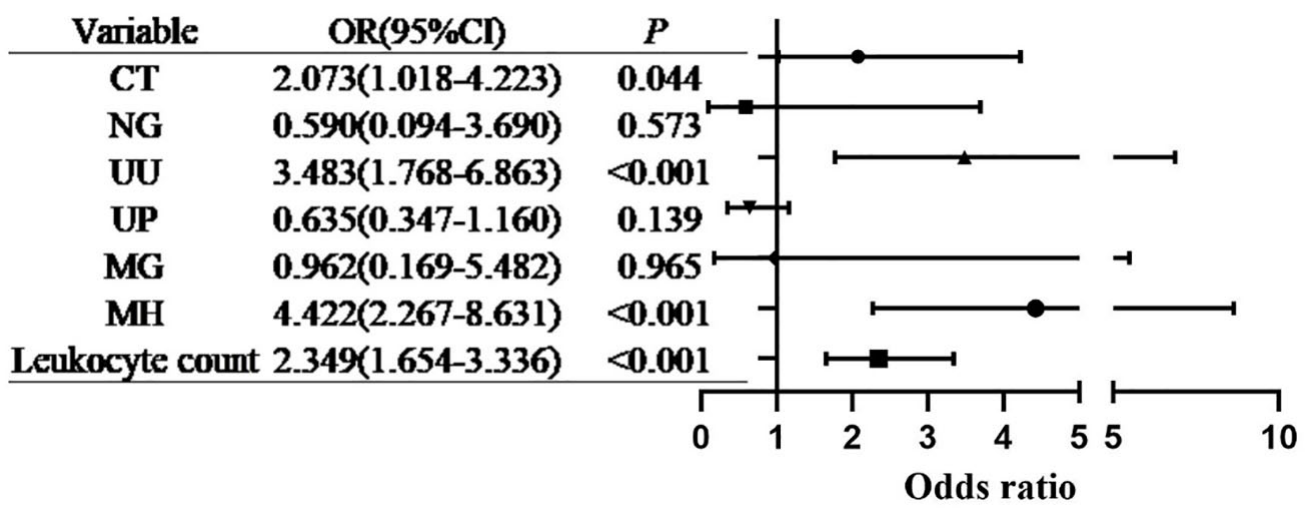
Multivariate logistic regression analysis of the associations between urogenital pathogens and BV (forest plot).

Univariate logistic regression analysis revealed that leukocyte count (OR = 1.660, 95% CI: 1.264–2.180, *P* < 0.001) was negatively associated with BV, whereas UU infection (OR = 1.994, 95% CI: 1.233–3.225, *P* = 0.005) and MH infection (OR = 2.607, 95% CI: 1.628–4.174, *P* < 0.001) were positively associated with BV. No significant associations were detected for CT, NG, UP, or MG infection (all *P* > 0.05) (**Table 4**, **Figure 5**).

**Table 4:** Univariate analysis of risk factors for BV.

Variables	*P*	Adjusted OR	OR (95%CI)
leukocyte count	< 0.001	1.660	1.264-2.180
*Chlamydia trachomatis*	0.218	1.382	0.825-2.318
*Neisseria gonorrhoeae*	0.250	0.394	0.080-1.927
*Ureaplasma urealyticum*	< 0.005	1.994	1.233-3.225
*Ureaplasma parvum*	0.672	1.103	0.700-1.739
*Mycoplasma hominis*	< 0.001	2.607	1.628-4.174
*Mycoplasma genitalium*	0.855	1.133	0.298-4.303

Multivariate logistic regression analysis identified several independent factors associated with BV. MH infection exhibited the strongest association (aOR = 4.422, 95% CI: 2.267–8.631, *P* < 0.001), followed by UU infection (aOR = 3.483, 95% CI: 1.768–6.863, *P* < 0.001), and CT infection (aOR = 2.073, 95% CI: 1.018–4.223, *P* = 0.044). Additionally, an elevated leukocyte count was significantly associated with BV status (aOR = 2.349, 95% CI: 1.654–3.336, *P* < 0.001). Pathogens such as NG, UP, and MG did not show significant associations in this multivariate model (all *P* > 0.05).

## 4 Discussion

According to the univariate analysis, several urogenital pathogens, including CT, NG, UU, UP, and MH, were significantly associated with HPV infection. An elevated leukocyte count and abnormal vaginal discharge were also significantly correlated with HPV infection. However, in the multivariate model, only CT infection, UU infection, elevated leukocytes, and abnormal vaginal discharge remained significant. In contrast, the associations of NG infection, UP infection, and MH infection were no longer statistically significant after adjustment, which may be attributed to the limited statistical power resulting from their low detection rates. This loss of significance after adjustment implies that their univariate associations were merely confounding artifacts driven by true independent factors, such as CT, UU, and vaginal inflammation.

CT, UU, UP, and MH infections were not only significantly associated with HPV but also closely related to the occurrence of vaginitis, potentially through disruption of the normal balance of the vaginal microbiota and promotion of inflammatory processes. Furthermore, the present study revealed that HPV infection itself is an independent risk factor for vaginitis, suggesting a possible bidirectional interaction. On the one hand, an inflammatory microenvironment may increase susceptibility to HPV; on the other hand, HPV infection may further exacerbate the dysbiosis of the vaginal microbiota, resulting in the formation of a positive feedback loop.

CT infection can induce epithelial-to-mesenchymal transition (EMT) in host cells, thereby facilitating tumorigenesis through loss of adhesion and suppression of DNA damage response pathways (Zadora et al., 2019). Several studies have suggested that persistent CT infection induces DNA damage via excessive generation of reactive oxygen species (ROS), thereby increasing the risk of carcinogenesis associated with high-risk human papillomavirus (hrHPV) (Knowlton et al., 2011; Sun et al., 2011; Zhu et al., 2016). Other investigations have indicated that CT may facilitate the acquisition of hrHPV infection by disrupting cadherin–catenin junctions in cervical epithelial cells (Prozialeck et al., 2002). In addition, the epidemiological evidence is inconsistent. Smith et al. reported a significant association between CT infection and squamous intraepithelial cervical cancer (Smith et al., 2004), whereas Castle et al. reported no correlation between CT infection and the severity of cervical neoplasia (Castle et al., 2003). Therefore, our results support that CT acts as a critical cofactor in HPV infection, likely by sustaining a pro-inflammatory microenvironment. Alongside the significant pathogenic role of CT, our multivariate analysis also highlighted UU as another crucial independent risk factor. Meta-analyses have shown that UU significantly increases the risk of overall HPV infection (OR≈1.57) and high-risk HPV infection (OR≈1.37) (Ye et al., 2018; Yang et al., 2024). Several studies have demonstrated an increased incidence of HPV coinfection in UU-infected women. The epidemiological is inconsistent, with some studies reporting significant associations but others reporting no such associations. Moreover, coinfections involving HPV, CT, UU, and MH have been frequently detected even in asymptomatic women, suggesting that polymicrobial coexistence is common in the general population (Camporiondo et al., 2016). A meta-analysis has further confirmed that BV, UU, and CT are significantly associated with HPV infection and CIN progression, whereas *Trichomonas vaginitis* and *vulvovaginal candidiasis* are not significantly associated with increased risks of HPV infection and CIN progression (Liang et al., 2019). The low prevalence of TV (n=1) and VVC (n=10) in our cohort precluded a robust evaluation of their independent effects on HPV. Therefore, the subsequent predictive modeling was directed specifically toward the more frequently observed urogenital coinfections.

In the univariate analysis, NG infection was significantly associated with HPV infection, suggesting that even with the low detection rate of NG in the present cohort, coinfection with NG may exacerbate cervical inflammation and increase susceptibility to HPV. The detection rates of CT, NG, and UU were significantly higher in the HPV-positive group (12.9%, 2.1%, and 77.5%, respectively) compared to the HPV-negative group (5.0%, 0.6%, and 62.3%, respectively; all P < 0.001) (Xuan et al., 2020).Furthermore, the positivity rates of CT, NG, and HPV increased with the severity of cervical lesions (Xu et al., 2022).MH infection was significantly associated with HPV infection and also closely linked to BV, indicating a potential role in promoting HPV persistence through disruption of the vaginal microenvironment.

MG is a well-established pathogen in pelvic inflammatory disease (PID), with a detection rate of 33.3% in women with acute PID, compared to just 6.7% in healthy controls (Zhang et al., 2010). Previous research has identified MG as an independent etiological agent of cervicitis (aOR ≈ 2.5) (Gaydos et al., 2009), and coinfection with CT and MG is common and may contribute to HPV infection. A multicenter Chinese study has reported an MG incidence of 3.8% among women with lower reproductive tract infections (Zhang et al., 2023). The latest Chinese Expert Consensus (2025) emphasized the independent pathogenicity of MG and its management importance (Zhang et al., 2025). Given its high recurrence rate and emerging antibiotic resistance, further studies are needed to clarify the role of MG in HPV persistence and cervical pathogenesis. However, in the present study, MG infection showed only a borderline association with HPV infection, a finding that may be attributable to the limited sample size of this cohort. In summary, NG, MG, and MH may act as cofactors in HPV acquisition and persistence, underscoring the importance of their screening and management in clinical practice.

Previous studies have shown that microbial dysbiosis contributes to cancer-related pathological processes by promoting inflammation and disrupting epithelial barrier integrity (Laniewski et al., 2020; Huang et al., 2024). Disturbances in the vaginal microbiota, particularly the depletion of Lactobacillus and the overgrowth of anaerobes, have been implicated in HPV acquisition and persistence (Watts et al., 2005; Gillet et al., 2011; Guo et al., 2012; Huang et al., 2024). BV is consistently associated with increased HPV risk and reduced clearance (Watts et al., 2005; Gillet et al., 2011; Guo et al., 2012). In the present study, UU, MH, and CT infection were significantly associated with BV, suggesting that sexually transmitted infections (STIs) may disrupt vaginal ecology and promote HPV persistence. These findings are consistent with those of epidemiological studies and meta-analyses (Camporiondo et al., 2016; Liang et al., 2019; Zhong et al., 2023; Yang et al., 2024), highlighting the importance of comprehensive screening and intervention for STIs and BV to maintain vaginal ecological stability.

Consistent with the findings of previous studies, CT and UU infection were independent risk factors for HPV infection, while an elevated leukocyte count and abnormal vaginal discharge also showed predictive value. Leukocytosis reflects increased local inflammation, impairing mucosal barriers and antiviral immunity (Laniewski et al., 2020; Huang et al., 2024). Shih et al. reported that increased WBC counts independently predict cervical neoplasia risk associated with high-risk HPV load and mediate the link between viral load and progression (Shih et al., 2024). Furthermore, Madeddu et al. reported that tumor-associated leukocytosis in patients with cervical cancer is correlated with poor outcomes, underscoring the impact of systemic inflammation on HPV-related malignancies (Madeddu et al., 2022). In line with these findings, Castle et al. reported that greater cervical inflammation among women with oncogenic HPV infection is associated with an increased risk of high-grade lesions (Castle et al., 2001). Obeagu (2025) emphasized that neutrophil-driven inflammation contributes critically to cervical carcinogenesis by promoting viral invasion (Obeagu, 2025). Mechanistically, Cheu et al. demonstrated that pathogen-induced neutrophil activation and extended survival result in the excessive release of reactive oxygen species and proteases (Cheu et al., 2022). These mediators disrupt epithelial intercellular junctions and compromise mucosal barrier integrity, thereby fostering a microenvironment conducive to HPV entry, persistence, and neoplastic progression. Collectively, these findings indicate that leukocyte count is both a marker of inflammation and systemic immune status, supporting its value in identifying women at risk of HPV infection and progression.

The proposed logistic regression model, which integrated CT infection, UU infection, leukocyte count, and clinical symptom data, demonstrated strong discrimination (AUC = 0.85) capability and good calibration, underscoring its potential utility for early clinical risk assessment. Beyond its statistical strength, the prediction model holds notable potential for clinical use. Current HPV screening strategies, primarily based on cytology and high-risk HPV testing, may overlook a subset of women at risk because of coinfections or inflammatory conditions. By incorporating these additional markers, the present model complements existing screening approaches by identifying these highly vulnerable women early. Crucially, our model is not intended to replace current screening modalities but to serve as a triage tool. By integrating stable microbial and inflammatory markers, this approach identifies which patients would benefit most from immediate colposcopy, thereby reducing unnecessary referrals and optimizing resource allocation—a strategy that is especially critical in low-resource healthcare systems. Thus, the predictive model may serve as a practical risk assessment tool to guide follow-up and preventive interventions.

Clinically, HPV persistence is defined as the consecutive detection of the same genotype over a 6- to 12-month longitudinal follow-up. Confined by its cross-sectional design, this study only reflects point-prevalence and cannot directly confirm persistence. However, the coinfections and local inflammation captured by our model are well-established key drivers of HPV persistence. Therefore, the high-risk population identified by our model essentially resides in a pathogenic microenvironment highly conducive to persistent HPV infection and subsequent progression to CIN.

The overall HPV positivity rate in the current study was notably high (46.4%), significantly surpassing the baseline prevalence reported for both the general Chinese population (12.1%–15.0%) and the global average (9.9%–11.7%). This elevated prevalence reflects the specific demographic of our hospital-based cohort. Recruited primarily from gynecological outpatient clinics, the study population inherently concentrated individuals presenting with urogenital symptoms, as evidenced by the 55.5% incidence of clinical vaginitis among participants. Given this inherent selection bias, our data represent a high-risk clinical demographic rather than a randomized community sample, thereby precluding direct extrapolation to the asymptomatic general population. Nevertheless, this symptomatic cohort accurately reflects real-world gynecological practice, making it highly valuable for studying the interactions between HPV and urogenital coinfections.

While high-throughput sequencing enables comprehensive microbial profiling, its high cost and lack of standardized clinical interpretation limit routine applicability. In contrast, targeted multiplex PCR offers superior analytical sensitivity and immediate clinical utility. Ma et al. (2022) characterized 29 HPV and 14 non-HPV STIs using NGS-STI, and Madhavan et al. (2026) demonstrated NGS utility in cervical cancer screening. Although our study employed targeted PCR rather than untargeted sequencing, the key pathogens identified align with high-risk microbial signatures reported in these sequencing cohorts.

The present study had several limitations. First, owing to the cross-sectional design, causal relationships among pathogen coinfections, inflammatory responses, and the acquisition or persistence of HPV could not be established. Second, certain behavioral and lifestyle factors, such as sexual activity frequency, contraceptive methods, and smoking history, were not systematically collected, which may introduce confounding effects on the observed associations. Although behavioral confounding (e.g., sexual exposure risk) cannot be fully decoupled in a cross-sectional study, evidence underscores a significant biological synergy. CT and UU may facilitate HPV acquisition and persistence by eliciting chronic localized inflammation and compromising the mucosal barrier. This aligns with the meta-analysis by Liang et al., identifying these pathogens as independent risk factors for CIN progression (Liang et al., 2019). Consequently, the synergistic effect of these co-infections warrants further attention in cervical cancer screening Crucially, the lack of HPV vaccination data represents a major unmeasure confounder. Third, the sample size for individuals with pathogens with low detection rates, such as NG and MG, was small, resulting in insufficient statistical power to fully evaluate their independent effects. Fourth, our molecular detection panel did not include PCR testing for *Candida* species. Although VVC was reliably diagnosed via microscopy, the number of VVC cases in our cohort was very limited compared to the high prevalence of BV. This small sample size restricts the statistical power to meaningfully evaluate the specific interaction between *Candida albicans* and HPV. Finally, the reliance on targeted PCR detection prevented comprehensive characterization of the vaginal microbiota, potentially leading to an underestimation of the complexity of the microbial dysbiosis. Future research should involve multicenter, large-scale prospective studies that incorporate high-throughput sequencing and multiomics approaches to validate and extend the findings of the present study.

## 5 Conclusion

In summary, the present study revealed CT infection and UU infection as independent risk factors for HPV infection, with an elevated leukocyte count and abnormal vaginal discharge serving as additional markers of localized inflammation. The model addresses a critical clinical gap in managing HPV-positive women. By identifying specific microbial signatures associated with viral infection, this diagnostic framework offers complementary insights to current protocols, enabling more precise risk stratification and timely intervention for those at risk of lesion progression. The predictive model may enhance existing HPV-screening protocols by serving as a cost-effective secondary triage tool, particularly in resource-limited settings. By resolving diagnostic ambiguities in cases characterized by fluctuating viral concentrations, the integration of STI co-infection markers enhances the identification of at-risk populations, thereby enabling more precise clinical management and refined longitudinal screening strategies. The generalizability of our model is anchored in the fundamental biological synergy between high-risk pathogens and HPV persistence; however, its robustness across diverse populations warrants further validation through large-scale, multi-center studies.

## Data Availability

The original contributions presented in the study are included in the article/supplementary material. Further inquiries can be directed to the corresponding author.

## References

[B1] Bao H. L. Jin C. Wang S. Song Y. Xu Z. Y. Yan X. J. . (2021). Prevalence of cervicovaginal human papillomavirus infection and genotypes in the pre-vaccine era in China: A nationwide population-based study. J. Infect. 82, 75–83. doi: 10.1016/j.jinf.2021.02.017. PMID: 33610682

[B2] Bruni L. Diaz M. Castellsagué X. Ferrer E. Bosch F. X. de Sanjosé S. . (2010). Cervical human papillomavirus prevalence in 5 continents: meta-analysis of 1 million women with normal cytological findings. J. Infect. Dis. 202, 1789–1799. doi: 10.1086/657321. PMID: 21067372

[B3] Camporiondo M. P. Farchi F. Ciccozzi M. Denaro A. Gallone D. Maracchioni F. . (2016). Detection of HPV and co-infecting pathogens in healthy Italian women by multiplex real-time PCR. Infez Med. 24, 12–17. 27031891

[B4] Castle P. E. Escoffery C. Schachter J. Rattray C. Schiffman M. Moncada J. . (2003). Chlamydia trachomatis, herpes simplex virus 2, and human T-cell lymphotrophic virus type 1 are not associated with grade of cervical neoplasia in Jamaican colposcopy patients. Sex Transm. Dis. 30, 575–580. doi: 10.1097/00007435-200307000-00009. PMID: 12838087

[B5] Castle P. E. Hillier S. L. Rabe L. K. Hildesheim A. Herrero R. Bratti M. C. . (2001). An association of cervical inflammation with high-grade cervical neoplasia in women infected with oncogenic human papillomavirus (HPV). Cancer Epidemiol. Biomarkers Prev. 10, 1021–1027. 11588127

[B6] Cheu R. K. Mohammadi A. Schifanella L. Broedlow C. Driscoll C. B. Miller C. J. . (2022). Altered innate immunity and damaged epithelial integrity in vaginal microbial dysbiosis. Front. Reprod. Health 4. doi: 10.3389/frph.2022.876729. PMID: 36303633 PMC9580658

[B7] de Sanjosé S. Diaz M. Castellsagué X. Clifford G. Bruni L. Muñoz N. . (2007). Worldwide prevalence and genotype distribution of cervical human papillomavirus DNA in women with normal cytology: a meta-analysis. Lancet Infect. Dis. 7, 453–459. doi: 10.1016/s1473-3099(07)70158-5. PMID: 17597569

[B8] Gaydos C. A. Maldeis N. E. Hardick A. Hardick J. Quinn T. C. (2009). Mycoplasma genitalium as a contributor to the multiple etiologies of cervicitis in women attending sexually transmitted disease clinics. Sex Transm. Dis. 36, 598–606. doi: 10.1097/olq.0b013e3181b01948. PMID: 19704398 PMC2924808

[B9] Gillet E. Meys J. F. Verstraelen H. Bosire C. De Sutter P. Temmerman M. . (2011). Bacterial vaginosis is associated with uterine cervical human papillomavirus infection: a meta-analysis. BMC Infect. Dis. 11, 10. doi: 10.1186/1471-2334-11-10. PMID: 21223574 PMC3023697

[B10] Guo Y. L. You K. Qiao J. Zhao Y. M. (2012). Bacterial vaginosis is conducive to the persistence of HPV infection. Int. J. STD AIDS 23, 581–584. doi: 10.1258/ijsa.2012.011342. PMID: 22930296

[B11] Huang R. Liu Z. Sun T. Zhu L. (2024). Cervicovaginal microbiome, high-risk HPV infection and cervical cancer: Mechanisms and therapeutic potential. Microbiol. Res. 287, 127857. doi: 10.1016/j.micres.2024.127857. PMID: 39121703

[B12] Knowlton A. E. Brown H. M. Richards T. S. Andreolas L. A. Patel R. K. Grieshaber S. S. (2011). Chlamydia trachomatis infection causes mitotic spindle pole defects independently from its effects on centrosome amplification. Traffic 12, 854–865. doi: 10.1111/j.1600-0854.2011.01204.x. PMID: 21477082 PMC3116664

[B13] Laniewski P. Ilhan Z. E. Herbst-Kralovetz M. M. (2020). The microbiome and gynaecological cancer development, prevention and therapy. Nat. Rev. Urol. 17, 232–240. doi: 10.1038/s41585-020-0286-z, PMID: 32071434 PMC9977514

[B14] Liang Y. Chen M. Qin L. Wan B. Wang H. (2019). A meta-analysis of the relationship between vaginal microecology, human papillomavirus infection and cervical intraepithelial neoplasia. Infect. Agent Cancer 14, 29. doi: 10.1186/s13027-019-0243-8. PMID: 31673281 PMC6815368

[B15] Ma Z. Gharizadeh B. Cai X. Li M. Fellner M. D. Basiletti J. A. . (2022). A comprehensive HPV-STI NGS assay for detection of 29 HPV types and 14 non-HPV sexually transmitted infections. Infect. Agent Cancer 17, 9. doi: 10.1186/s13027-022-00420-8. PMID: 35313939 PMC8935747

[B16] Madeddu C. Deidda M. Panzone F. Tanca L. Cherchi M. C. Scartozzi M. . (2022). Pathogenic and prognostic roles of paraneoplastic leukocytosis in cancer. Cancers (Basel) 14, 3964. doi: 10.3390/diagnostics12081910. PMID: 36010957

[B17] Madhavan Y. Muthukumar V. Ramasubramanian S. Ramshankar V. (2026). High throughput HPV genotyping by next generation sequencing for detection of 28 HPV types and 13 sexually transmitted infections: A first community-based cervical cancer screening study from India. Diagn. Microbiol. Infect. Dis. 114, 117278. doi: 10.1016/j.diagmicrobio.2026.117278. PMID: 41576634

[B18] Obeagu E. I. (2025). From inflammation to invasion: neutrophils in cervical cancer pathogenesis. Ann. Med. Surg. (Lond) 87, 7156–7171. doi: 10.1097/ms9.0000000000002679. PMID: 41180630 PMC12577824

[B19] Prozialeck W. C. Fay M. J. Lamar P. C. Pearson C. A. Sigar I. Ramsey K. H. (2002). Chlamydia trachomatis disrupts N-cadherin-dependent cell-cell junctions and sequesters β-catenin in human cervical epithelial cells. Infect. Immun. 70, 2605–2613. doi: 10.1128/iai.70.5.2605-2613.2002. PMID: 11953402 PMC127927

[B20] Shih Y. W. Wang C. Y. Liu Y. H. Chang C. L. Lin M. H. Tsai H. T. (2024). Increased white blood cell count mediates the association between high-risk human papillomavirus DNA load, tobacco exposure and cervical neoplasia risk. J. Pers. Med. 14, 178. in Chinese. 38392611

[B21] Smith J. S. Bosetti C. Muñoz N. Herrero R. Bosch F. X. Eluf-Neto J. . (2004). Chlamydia trachomatis and invasive cervical cancer: A pooled analysis of the IARC multicentric case-control study. Int. J. Cancer 111, 431–439. doi: 10.1002/ijc.20257. PMID: 15221973

[B22] Sun H. S. Wilde A. Harrison R. E. (2011). Chlamydia trachomatis inclusions induce asymmetric cleavage furrow formation and ingression failure in host cells. Mol. Cell. Biol. 31, 5011–5022. doi: 10.1128/mcb.05734-11. PMID: 21969606 PMC3233033

[B23] Watts D. H. Fazzari M. Minkoff H. Hillier S. L. Sha B. Glesby M. . (2005). Effects of bacterial vaginosis and other genital infections on the natural history of human papillomavirus infection in HIV-1-infected and high-risk HIV-1-uninfected women. J. Infect. Dis. 191, 1129–1139. doi: 10.1086/427777. PMID: 15747249

[B24] Xu Y. R. Lin J. T. Zhou Z. W. Xuan C. Qiu C. L. (2022). Correlation of CT and NG infections with HPV infection and cervical lesions. J. Mol. Diagn. Ther. 14, 2215–2218. in Chinese.

[B25] Xuan B. B. Tan M. Y. Sun H. X. Sheng H. M. (2020). Analysis of co-infections of human papillomavirus with Ureaplasma urealyticum, Chlamydia trachomatis, and Neisseria gonorrhoeae in Changning District, Shanghai. Lab. Med. 35, 859–863. in Chinese.

[B26] Yang J. Long X. Li S. Zhou M. Hu L. N. (2024). The correlation between vaginal pathogens and high-risk human papilloma virus infection: a meta-analysis of case-control studies. Front. Oncol. 14. doi: 10.3389/fonc.2024.1423118. PMID: 39640283 PMC11618108

[B27] Yang L. Long X. Li Z. Zhou Y. Hu Y. (2024). The correlation between vaginal pathogens and high-risk human papillomavirus (HR-HPV) infection: a meta-analysis. Infect. Agent Cancer 19. doi: 10.3389/fonc.2024.1423118. PMID: 39640283 PMC11618108

[B28] Ye H. Song T. Zeng X. Li L. Hou M. Xi M. . (2018). Association between genital mycoplasmas infection and cervical HPV infection, abnormal cervical cytopathology, and cervical cancer: a systematic review and meta-analysis. Arch. Gynecol Obstet. 297, 1411–1429. doi: 10.1007/s00404-018-4733-5. PMID: 29520664

[B29] Zadora P. K. Chumduri C. Imami K. Berger H. Mi Y. Selbach M. . (2019). Integrated phosphoproteome and transcriptome analysis reveals Chlamydia-induced epithelial-to-mesenchymal transition in host cells. Cell Rep. 26, 1286–1302, e1288. doi: 10.2139/ssrn.3151992. PMID: 30699355

[B30] Liu Z. H. Zhang D. Hong K. Yang L. G. Yuan Y. M. He H. . (2025). Chinese expert consensus on the diagnosis and treatment of genital mycoplasma infections (2025 edition). Chin. J. Pract. Gynecology Obstetrics 41, 541–547. doi: 10.1097/cm9.0000000000003172. PMID: 38863122 PMC11230803

[B31] Zhang D. Wei H. Liao Q. P. Ma J. M. (2010). Detection rate of Mycoplasma and other pathogens in female reproductive tract in acute pelvic inflammatory disease. Chin. J. Maternal Child Health 25, 366–369. in Chinese.

[B32] Zhang Z. Zong X. Bai H. Fan L. Li T. Liu Z. (2023). Prevalence of Mycoplasma genitalium and Chlamydia trachomatis in Chinese female with lower reproductive tract infection: a multicenter epidemiological survey. BMC Infect. Dis. 23, 2. doi: 10.1186/s12879-022-07975-2. PMID: 36604611 PMC9814310

[B33] Zhong C. Li X. Teng Y. Tian J. (2023). Co-infection with human papillomavirus and sexually transmitted infections among Chinese individuals. Microb. Pathog. 185, 106395. doi: 10.1016/j.micpath.2023.106395. PMID: 37852554

[B34] Zhu H. Shen Z. Luo H. Zhang W. Zhu X. (2016). Chlamydia trachomatis infection-associated risk of cervical cancer: a meta-analysis. Medicine 95, e3077. doi: 10.1097/md.0000000000003077. PMID: 27043670 PMC4998531

